# Eyelid Ptosis as an Atypical Manifestation of Meningeal Cryptococcosis: A Case Report

**DOI:** 10.7759/cureus.93907

**Published:** 2025-10-05

**Authors:** Rosa Sá, Hugo Goncalves, Bárbara Fraga Campos, Francisco De Oliveira Simões, Rui M Domingues, Narciso Oliveira, Teresa Pimentel

**Affiliations:** 1 Division of Oncology, Unidade Local de Saúde de Braga, Braga, PRT; 2 Division of Rheumatology, Unidade Local de Saúde de Braga, Braga, PRT; 3 Division of Internal Medicine, Unidade Local de Saúde de Braga, Braga, PRT

**Keywords:** antiretroviral therapy, cryptococcal meningitis, hiv, opportunistic infections, ptosis

## Abstract

Cryptococcal meningitis remains a significant opportunistic infection in people living with human immunodeficiency virus (HIV), occurring with advanced immunosuppression.

We report the case of a 59-year-old HIV-type-1-positive man, previously with an undetectable viral load and a CD4+ count of 504 cells/μL, who presented with altered mental status and right-sided ptosis. He had missed follow-up for one year, and repeat testing showed a CD4+ count of 218 cells/μL and a viral load of 258,000 copies/mL. Brain MRI revealed leptomeningeal enhancement of the basal cisterns. Cerebrospinal fluid analysis confirmed *Cryptococcus* spp. The patient completed induction therapy with amphotericin B and flucytosine, with marked neurological improvement. Antiretroviral therapy (ART) was resumed after stabilization, and consolidation therapy with fluconazole was initiated.

This case highlights an atypical presentation of cryptococcal meningitis, with CD4+ count above 100 cells/μL, and underscores the importance of ART adherence and prompt investigation of subacute neurological symptoms in HIV-infected individuals.

## Introduction

Cryptococcal meningitis is an opportunistic infection primarily caused by *Cryptococcus neoformans*, predominantly affecting immunocompromised individuals, especially those living with human immunodeficiency virus (HIV) [1,2].

Despite a reduced incidence in developed countries due to earlier diagnosis and widespread access to antiretroviral therapy (ART), it remains a significant cause of morbidity and mortality among this population. The classic clinical presentation includes headache, fever, neck stiffness, and neurological disturbances, typically occurring in the context of advanced immunosuppression, most commonly with CD4+ T-cell counts below 100 cells/µL. Nevertheless, cases have been documented in patients with preserved immune function, including normal CD4+ counts, highlighting the variable clinical spectrum of the disease. Atypical presentations can further delay diagnosis and treatment [1,2].

Neurological manifestations of cryptococcal disease include meningitis, myelitis, encephalitis, and cryptococcoma. Isolated eyelid ptosis is a rare initial manifestation of cryptococcal meningoencephalitis and is indicative of cranial nerve involvement, with only a few cases described in the literature [1-3].

We report a unique case of unilateral eyelid ptosis as the initial clinical sign of cryptococcal meningitis in an HIV-positive patient with a CD4+ T-cell count above 200 cells/µL.

## Case presentation

A 59-year-old man, previously autonomous in daily activities, presented to the emergency department with altered mental status and progressive fatigue over the preceding days. He was found collapsed at home. He denied fever, headache, or other focal complaints. On physical examination, he appeared cachectic and dehydrated, although mucous membranes were well perfused. Cardiac and pulmonary auscultation were unremarkable. Neurological examination revealed confusion and isolated right-sided ptosis.

His medical history was notable for HIV-type-1 infection diagnosed 25 years prior, gait ataxia, bilateral severe hearing loss due to tympanosclerosis, active tobacco and alcohol use, and a previous episode of pulmonary and lymph node tuberculosis in 2011. Laboratory results showed leukocytosis and neutrophilia (17,400 leukocytes/μL, 13,900 neutrophils/μL), as well as acute kidney injury (urea 58 mg/dL, creatinine 1.8 mg/dL). Urinalysis and remaining blood tests were unremarkable (Table 1). A non-contrast cranial CT scan showed no acute changes. He was admitted for further investigation.

**Table 1:** Laboratory values at admission. WBC: white blood cells; BUN: blood urea nitrogen; AST: aspartate aminotransferase; ALT: alanine aminotransferase; ALP: alkaline phosphatase; LDH: lactate dehydrogenase; CRP: C-reactive protein; HIV: human immunodeficiency virus

	Result	Reference range
Hemoglobin	17 g/dL	13.5-17 g/dL
Hematocrit	50.9%	40.0-49.5%
WBC	17,400/µL	4,000-11,000/µL
Neutrophils	13,900/µL	1,800-7,100/µL
Platelets	168,000/µL	150,000-400,000/µL
BUN	58 mg/dL	19-49 mg/dL
Creatinine	1.8 mg/dL	0.7-1.2 mg/dL
Potassium	4.6 mmol/L	3.5-5.1 mmol/L
Sodium	136 mmol/L	136-145 mmol/L
Total bilirubin	0.8 mg/dL	0.3-1.2 mg/dL
AST	38 U/L	12-40 U/L
ALT	22 U/L	7-40 U/L
ALP	75 U/L	46-116 U/L
LDH	240 U/L	120-246 U/L
CRP	7.6 mg/L	<5.0 mg/L
CD4+ T-cell count	218 cells/µL	500-1,200 cells/µL
HIV viral load	258,000 copies/mL	<50 copies/mL

Review of clinical records revealed that the patient had missed multiple HIV follow-up appointments over the past year, despite having previously demonstrated adequate adherence to ART. His last documented CD4+ T-cell count had been 504 cells/μL with an undetectable viral load. During hospitalization, his gait ataxia worsened, and he developed episodes of confusion. Repeat testing revealed a CD4+ count of 218 cells/μL and a viral load of 258,000 copies/mL (Table 1).

**Figure 1:**
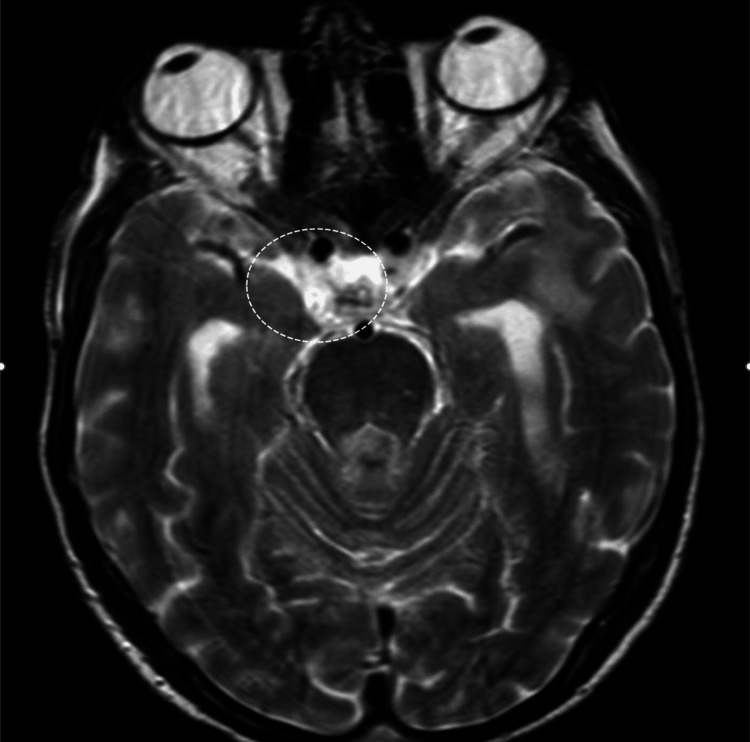
Brain MRI showing leptomeningitis with diffuse leptomeningeal enhancement and pachymeningeal enhancement extending into the right cavernous sinus.

Brain MRI was performed and revealed leptomeningeal enhancement in the basal cisterns, raising the suspicion of central nervous system tuberculosis (Figure 1).

A lumbar puncture was performed, yielding clear cerebrospinal fluid (CSF) with lymphocytic pleocytosis and reduced glucose. Opening pressure was 21 cmH₂O. India ink staining was positive for *Cryptococcus *spp., confirmed by cryptococcal antigen testing. CSF culture was positive for *Cryptococcus neoformans.* It was negative for mycobacteria and John Cunningham (JC) virus (Table 2).

**Table 2:** Cerebrospinal fluid analysis. PCR: polymerase chain reaction; JC: John Cunningham

	Result	Reference range
Appearance	Clear	Clear
Opening pressure	21 cmH₂O	6-20 cmH₂O
Cell count	341 cells/µL	0-5 cells/µL
Lymphocytes	88%	60-70%
Glucose	14 mg/dL	40-70 mg/dL
Protein	3.29 g/L	0.15-0.45 g/L
Lactate	5.5 mmol/L	0.6-2.2 mmol/L
India ink stain	Positive	-
Cryptococcal antigen	Positive	-
Mycobacteria real-time PCR and culture	Negative	-
JC virus PCR	Negative	-

Induction therapy with amphotericin B and flucytosine was initiated and maintained for 18 days. Two additional lumbar punctures were performed during hospitalization, with the last showing negative results for *Cryptococcus*. The patient demonstrated significant clinical improvement, including the normalization of consciousness and resolution of ptosis. ART was resumed, and consolidation therapy with fluconazole was initiated.

## Discussion

Cryptococcal meningitis is a life-threatening opportunistic infection caused predominantly by *Cryptococcus neoformans*, with a particular predilection for immunocompromised hosts, especially individuals with advanced HIV infection. The risk of infection correlates strongly with a decline in CD4+ T-cell count, typically occurring in patients with counts below 100 cells/μL [1]. Nevertheless, there have been reports of cases occurring in patients with relatively preserved immune function, including CD4+ counts above 200 cells/μL, as observed in this case [2,3].

Our patient presented with an atypical initial manifestation (isolated unilateral ptosis) prior to the development of more classic neurological symptoms such as confusion. Isolated cranial nerve involvement is a rare presentation of cryptococcal meningoencephalitis and is believed to result from direct fungal invasion or the inflammation of the basal meninges, leading to the compression or infiltration of cranial nerves, particularly in the region of the basal cisterns [4]. MRI findings in this case, demonstrating leptomeningeal enhancement in the basal cisterns, supported this mechanism.

The initial normal CD4+ count and undetectable viral load underscore the importance of clinical vigilance. Although the patient's last known immunological status was stable, poor ART adherence (suspected based on missed follow-ups and subsequent laboratory evidence of viral rebound and CD4+ decline) likely contributed to the immunosuppression that precipitated disease reactivation. This reflects the dynamic nature of HIV infection, where even short-term lapses in ART adherence can result in significant immune deterioration [5].

Diagnosis was established through CSF analysis, which revealed lymphocytic pleocytosis, hypoglycorrhachia, and a positive India ink stain and positive cryptococcal antigen testing [6]. Imaging played a crucial role in raising early suspicion, particularly given the atypical presentation. Brain MRI has greater sensitivity than CT for detecting meningeal involvement and is essential in evaluating for complications such as hydrocephalus or cryptococcomas [7].

The management of cryptococcal meningitis in HIV-infected patients consists of a three-phase approach: induction with amphotericin B plus flucytosine for at least two weeks, consolidation with high-dose fluconazole for eight weeks, and maintenance with lower-dose fluconazole until immune reconstitution is achieved [7]. In our case, the patient completed an 18-day induction phase and showed clinical improvement, including the resolution of ptosis and recovery of consciousness.

Given the high risk of immune reconstitution inflammatory syndrome (IRIS), particularly when ART is initiated or resumed soon after antifungal therapy, guidelines recommend deferring ART reintroduction by four to six weeks in patients with cryptococcal meningitis [8]. Our patient's ART was deferred during antifungal treatment and successfully resumed after initial clinical improvement.

## Conclusions

This case highlights that cryptococcal meningitis can occur even in patients with relatively high CD4+ counts for an opportunistic infection. Isolated cranial nerve palsies, such as unilateral ptosis, are rare but important early signs that should raise suspicion for central nervous system infections, particularly in individuals with HIV. Early neuroimaging and prompt lumbar puncture are crucial for diagnosis, especially in atypical presentations, as the timely detection of leptomeningeal involvement can guide urgent treatment decisions.

This report also underscores the importance of strict ART adherence and regular follow-up to prevent opportunistic infections. Even brief lapses in treatment can lead to rapid immune decline and the reactivation of latent infections. Clinicians should maintain a high index of suspicion for cryptococcal meningitis in HIV-positive patients with subacute neurological symptoms, regardless of recent laboratory results, and carefully time ART reintroduction to reduce the risk of immune reconstitution inflammatory syndrome.
